# Left hemispheric dominance during auditory processing in a noisy environment

**DOI:** 10.1186/1741-7007-5-52

**Published:** 2007-11-15

**Authors:** Hidehiko Okamoto, Henning Stracke, Bernhard Ross, Ryusuke Kakigi, Christo Pantev

**Affiliations:** 1 Institute for Biomagnetism and Biosignalanalysis, University of Muenster, Muenster, Germany; 2 Rotman Research Institute at Baycrest Centre, University of Toronto, Ontario, Canada; 3 Department of Integrative Physiology, National Institute for Physiological Sciences, Okazaki, Japan

## Abstract

**Background:**

In daily life, we are exposed to different sound inputs simultaneously. During neural encoding in the auditory pathway, neural activities elicited by these different sounds interact with each other. In the present study, we investigated neural interactions elicited by masker and amplitude-modulated test stimulus in primary and non-primary human auditory cortex during ipsi-lateral and contra-lateral masking by means of magnetoencephalography (MEG).

**Results:**

We observed significant decrements of auditory evoked responses and a significant inter-hemispheric difference for the N1m response during both ipsi- and contra-lateral masking.

**Conclusion:**

The decrements of auditory evoked neural activities during simultaneous masking can be explained by neural interactions evoked by masker and test stimulus in peripheral and central auditory systems. The inter-hemispheric differences of N1m decrements during ipsi- and contra-lateral masking reflect a basic hemispheric specialization contributing to the processing of complex auditory stimuli such as speech signals in noisy environments.

## Background

In most day to day situations, we are exposed to many different sound inputs simultaneously. During encoding and perception, these concurring sound inputs interact with each other. A well known phenomenon in this regard is the elevation of the hearing threshold for a test sound in presence of a competing sound. This phenomenon, called 'simultaneous masking' [1], can be divided into two categories depending on whether test signal and competing sound are presented to the same ear ('ipsi-lateral masking') or to different ears ('contra-lateral masking'). In humans, simultaneous masking has been investigated in a series of psychoacoustical experiments [2]. However, the underlying neurophysiological mechanisms remain unclear.

In the case of ipsi-lateral masking, interactions already take place at the cochlear level, thus this type of masking is often referred to as 'peripheral masking' [3]. The traveling wave induced by a test signal overlaps with the deflection pattern produced by the masker on the basilar membrane. Thus, the simultaneously presented masker distorts the auditory nerve discharges elicited by the test signal. A magnetoencephalography (MEG) study [4] investigated the auditory evoked responses elicited by the test tone presented together with continuous band-eliminated noises characterized by different eliminated frequency sections centered around test tone frequency during ipsi-lateral masking. Band-eliminated noises with relatively narrow eliminated bands caused smaller N1m (the most prominent negative deflection of the slow auditory evoked field) responses as compared to band-eliminated noises with wider eliminated bands; the N1m response was interpreted as reflection of the ipsi-lateral masking effect.

During contra-lateral simultaneous masking, signal and masker are presented to opposite ears; therefore, interactions can only take place in the central auditory pathway. Thus, this type of masking is referred to as 'central masking' [3]. The threshold shift produced by contra-lateral masking is much smaller compared to ipsi-lateral masking. A maximal shift was observed when a tonal masker similar in frequency to the test tone was presented to the contra-lateral ear [5]. An electroencephalographic experiment [6] showed that the auditory steady state response (ASSR), which is considered to have a primary auditory cortex origin [7,8], became smaller when contra-laterally presented continuous maskers became louder. These results indicated that contra-lateral masking effects can be observed also in primary auditory cortex. However, an MEG study [9] showed that contra-laterally presented continuous noise did not cause a significant N1m decrement in response to the test tone. Both studies adopted similar broadband noises as maskers, but test stimuli differed. In the former study, 0.1 ms square pulses at a rate of 39 Hz were presented, whereas the latter study used a 500 Hz square wave tone. Hence, the different contra-lateral masking effects observed between studies might have been caused by the different generators of ASSR (in the primary auditory cortex; [7,10,11]) and the N1m (in lateral aspects of Heschl's gyrus and the posterior temporal plane; [7,12]) as well as by the different spectral and temporal features of the test stimuli.

Both spectral and temporal features of test stimuli and maskers might play an important role for simultaneous masking effects. In a psychoacoustical experiment, Fletcher [13] presented a test pure tone stimulus simultaneously with a narrow-band noise characterized by a pass-band centered at the pure tone frequency. The hearing threshold for the test tone increased until the pass-band of the narrow-band noise reached a certain bandwidth (the 'critical band'): beyond that bandwidth, the hearing threshold remained constant. Fletcher concluded that only those parts of the noise close in spectral content to the test tone frequency could have contributed to the elevation of the hearing threshold irrespective of temporal sound features. However, temporal features of sound signals might also influence the simultaneous masking effect [14].

Recent neuroimaging techniques revealed functional hemispheric asymmetries of spectral and temporal neural processing. A left hemispheric dominance for temporal processing and a right hemispheric dominance for spectral processing were observed by means of positron emission tomography (PET; [15]) and functional magnetic resonance imaging (fMRI; [16]). In psychoacoustical experiments [2,14], it has been shown that both temporal and spectral processing are important for the encoding of the test stimulus during simultaneous masking. Thus, according to the results of PET and fMRI [15,16], simultaneous masking effects might differ between left and the right hemispheres. However, the exact underlying neural mechanisms and the hemispheric differences related to simultaneous masking remain elusive.

The goals of the present study were: (1) to investigate interactions between auditory neurons characterized by similar or different receptive fields during both ipsi- and contra-lateral masking, and (2) to investigate the hemispheric differences in neural activities in human auditory cortex during both ipsi- and contra-lateral masking by means of MEG. The results of this study were expected to yield new information about underlying neural mechanisms and hemispheric differences for the processing of complex sound stimuli during simultaneous masking.

## Results

**Figure 1:**
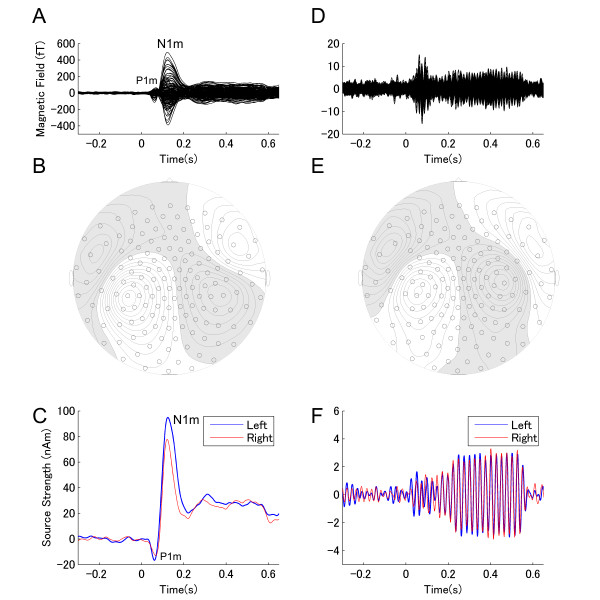
**Representative single subject results**. (A) Overlay of individual magnetic field waveforms of all channels (30 Hz low-pass filtered). (B) The contour map of the magnetic field distribution for the maximal N1m response at the latency of 0.118 s. (C) The cortical source strength obtained from the source space projection approach applied to the magnetic field waveforms in (A). Blue and red lines represent the source strengths in the left and right hemispheres, respectively. (D) Overlay of individual magnetic field waveforms of all channels representing the auditory steady state response (ASSR; band-pass filtered between 30 to 50 Hz). (E) The contour map of the magnetic field distribution at the maximum field distribution at the latency of 0.337 s. (F) The cortical source strength obtained from the source space projection approach applied to the magnetic field waveforms (D).

An example of individual magnetic field waveforms (30 Hz low-pass filtered) for the no masking condition (Figure 1A) demonstrates the N1m response peaking approximately 100 ms after the onset of the test stimulus (TS) as most pronounced component of the auditory evoked fields. P1m waves (preceding the N1m) were also visible, but small and variable across subjects. Thus, in the present study, we focused on the N1m response. The source waveforms also exhibited the P1m-N1m response complex to the onset of the TS (Figure 1C). An example of individual magnetic field waveforms (same subject) for the ASSR (band-pass filtered between 30 to 50 Hz) for the no masking condition is displayed in Figure 1D. The signals exhibit the transient evoked gamma-band response and the development of the ASSR after TS onset (Figure 1D,F). The waveforms show clear polarity reversal. Even though the field amplitudes were smaller compared to the N1m, the iso-contour plots of the magnetic field distribution demonstrates a pattern typically resulting from dipolar sources (Figure 1B,E).

**Figure 2:**
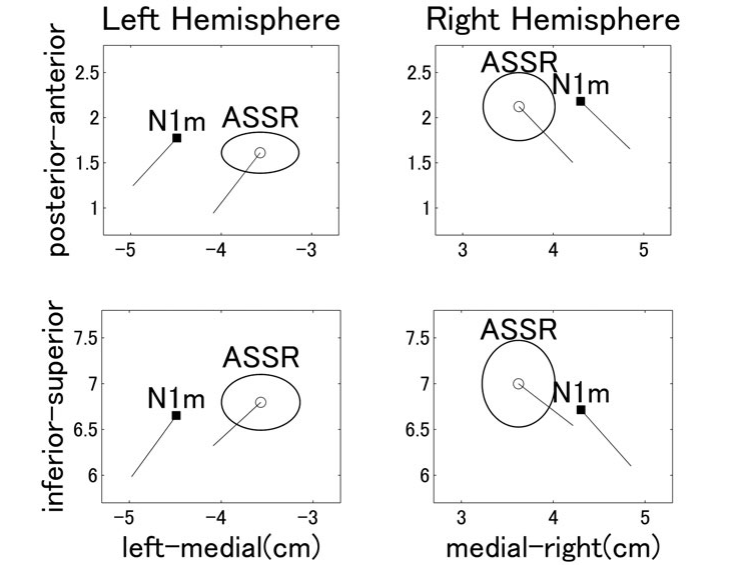
**Source locations**. Grand averaged localization of N1m sources (square filled symbols) and ASSR sources (open circles) in the y-x plane (medial-lateral direction vs posterior-anterior direction, upper graph) and y-z plane (medial-lateral direction vs inferior-superior direction, lower graph). The solid line starting at each dipole location represents the mean orientation of the equivalent current dipole. The ellipses around ASSR dipole locations denote the 95% confidence intervals for the distance between ASSR and N1m sources.

The goodness of fit of the equivalent current dipoles (ECDs) for N1m and ASSR in the control condition was above 90% for all participants. The grand averaged ECD source locations (Figure 2) in y-z-plane (medial-lateral and inferior-superior directions) and y-x-plane (medial-lateral and posterior-anterior directions) demonstrated a significant separation between N1m and ASSR sources in the medial-lateral (y) direction (left hemisphere: t(9) = 4.01, p < 0.01, right hemisphere: t(9) = 2.96, p = 0.016). In both hemispheres, ASSR sources were located significantly more medial than N1m sources, and both ASSR and N1m sources were located more anterior in the right hemisphere (N1m: t(9) = 2.39, p = 0.041, ASSR: t(9) = 2.19, p = 0.056).

**Figure 3:**
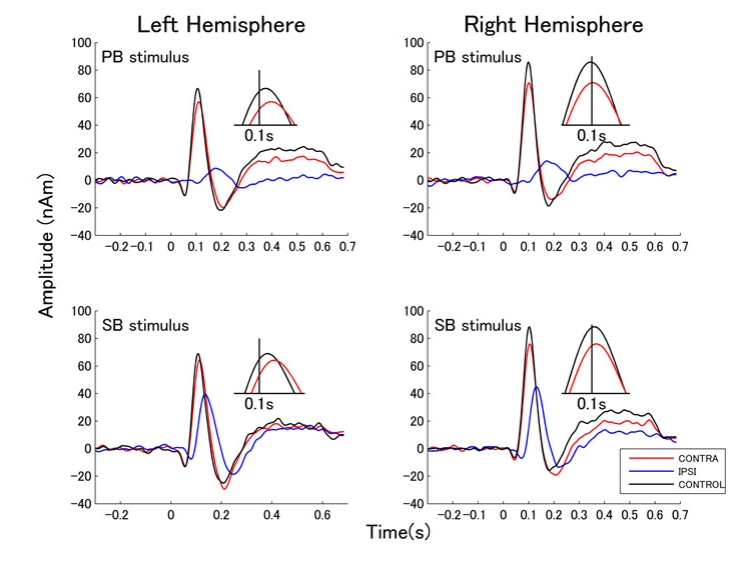
**Source waveforms elicited by left ear stimulation**. Grand averages of source space projection waveforms for left ear stimulation. The upper graphs show the responses to the PB. The lower graphs show the responses to SB. Left and right graphs denote the responses from left and right hemisphere. The responses elicited by different conditions are represented by different colored lines (see box in the right lower corner). The insets show the peaks of the N1m responses for the contra-lateral and the control condition on an enlarged time scale around a latency of 0.1 s. As the N1m responses during ipsi-lateral masking were small and delayed, they are not shown in the insets.

**Figure 4:**
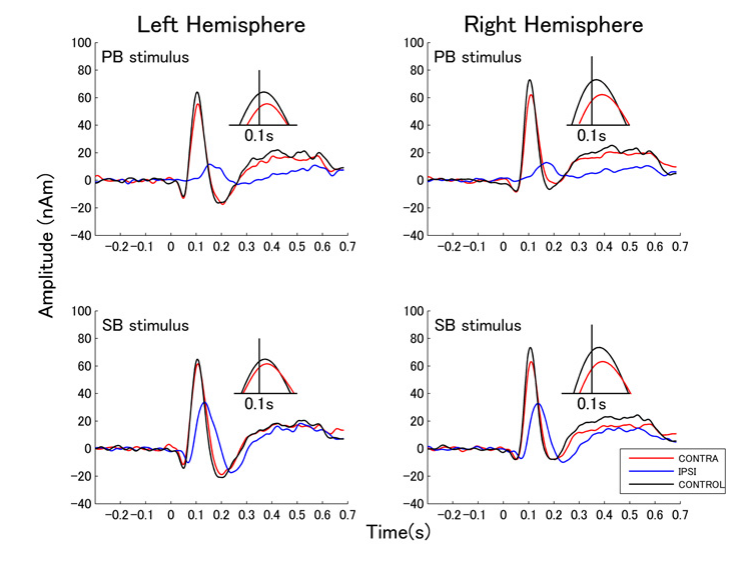
**Source waveforms elicited by right ear stimulation**. Grand averages of dipole moment waveforms for right ear stimulation (arrangement according to Figure 3).

The grand averaged source strength waveforms elicited by TS presented to left and right ears for each masking condition across all subjects are shown in Figures 3 and 4, respectively. The figures exhibit the P1m-N1m response complexes to the onset of the TS for the contra-lateral and the control condition. In case of ipsi-lateral masking, N1m responses were small and delayed.

### Ipsi-lateral masking

**Figure 5:**
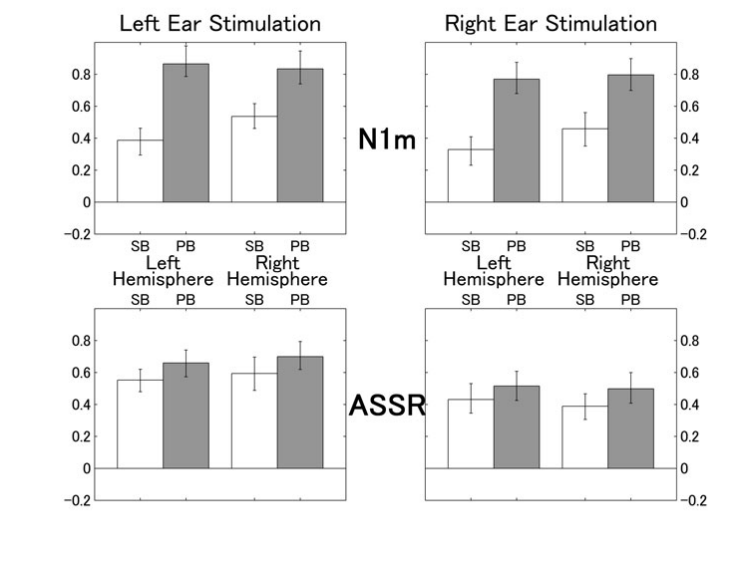
**Normalized source strength decrements for ipsi-lateral masking**. Normalized N1m and ASSR source strength decrements for the ipsi-lateral condition (normalized decrement = (source strength_unmasked_-source strength_ipsi-lateral_)/source strength_unmasked_) representing the masking effects between 0 (no masking) and 1 (complete extinction). The upper graphs denote the normalized N1m decrements and the lower graphs denote the normalized ASSR decrements for the left and the right hemisphere during left (left column) or right ear (right column) stimulation. The error bars denote the 95% confidence limits.

The means of the normalized N1m and ASSR source strength decrements obtained in the ipsi-lateral condition are displayed in Figure 5 with lower and upper 95% confidence limits. A repeated measures analysis of variance (ANOVA) applied to normalized N1m source strength decrements during ipsi-lateral masking resulted in significant main effects for TS-TYPE (F (1, 9) = 179, p < 0.0001) and HEMISPHERE (F (1, 9) = 12.3, p < 0.01), as well as a significant interaction between TS-TYPE and HEMISPHERE (F (1, 9) = 6.6, p = 0.030). The stimulation side had no effect on these significant differences. These findings indicate that larger normalized N1m decrements were obtained for the pass-band stimulus (PB) compared to the stop-band stimulus (SB), and for the right hemisphere compared to the left. This result means that the ipsi-lateral masking effect on the N1m was more effective when stimulus and masker shared spectral content, and less effective when stimulus and masker were different in spectrum. In addition, the ipsi-lateral masking effect on N1m was stronger in the right hemisphere, and this hemispheric difference was independent of stimulation side. The significant interaction between TS-TYPE and HEMISPHERE shows that this hemispheric difference was mainly caused by SB.

A repeated measures ANOVA applied to normalized ASSR source strength decrements during ipsi-lateral masking resulted in a significant main effect for TS-TYPE (F (1, 9) = 14.3, p < 0.01) and STIMULATION-SIDE (F (1, 9) = 11.3, p < 0.01); however, neither a significant main effect for HEMISPHERE nor an interaction was found. These findings indicate that similar N1m and ASSR decrement patterns were obtained for TS-TYPE. However, contrary to the N1m decrement pattern, ASSR decrements did not show a significant hemispheric difference, but a significant difference for STIMULATION-SIDE. This result means that the ipsi-lateral masking effect on the ASSR was stronger when stimulus and masker were presented to the left ear, and this stimulation-side difference was not hemisphere specific.

### Contra-lateral masking

**Figure 6:**
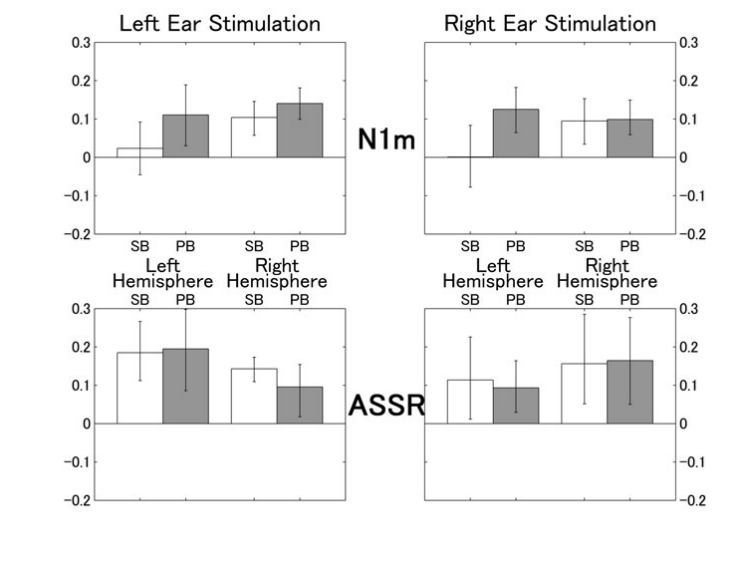
**Normalized source strength decrements for contra-lateral masking**. Normalized N1m and ASSR source strength decrements for contra-lateral masking (graphs are assorted according to Figure 5).

The means of the normalized N1m and ASSR source strength decrements across subjects for the contra-lateral masking condition are presented in Figure 6. A repeated measures ANOVA applied to the normalized N1m source strength decrements during contra-lateral masking resulted in significant main effects for TS-TYPE (F (1, 9) = 7.8, p = 0.021) and HEMISPHERE (F (1, 9) = 6.0, p = 0.036), as well as a significant interaction between TS-TYPE and HEMISPHERE (F (1, 9) = 8.0, p = 0.020). These findings indicated that the N1m decrement pattern observed during contra-lateral masking was identical to the N1m decrement pattern observed during ipsi-lateral masking, even though the masking effects during contra-lateral masking were far smaller than during ipsi-lateral masking. Contra-lateral masking effects were larger for the test sound of similar frequency and for the right hemisphere. This hemispheric difference was independent of stimulation side.

The normalized ASSR decrement during contra-lateral masking was significantly different from zero (no masking effect) in all conditions (Figure 6). This means that the ASSR was significantly smaller during contra-lateral masking compared to the control condition, even though the contra-lateral masking effect was far smaller compared to ipsi-lateral masking. A repeated measures ANOVA applied to the ASSR ratios during contra-lateral masking revealed neither a significant main effect nor a significant interaction.

### Threshold shift

**Figure 7:**
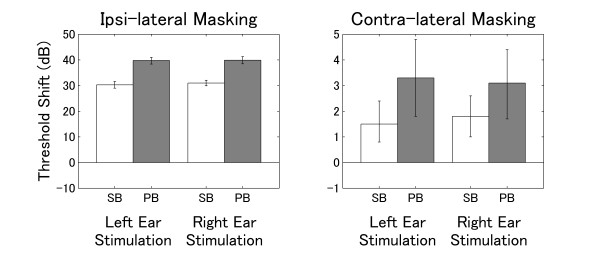
**Threshold shifts**. Threshold shifts during ipsi- and contra-lateral masking. The left graph denotes the threshold shift during ipsi-lateral masking, the right graph denotes the shift during contra-lateral masking. The error bars represent the 95% confidence limits for the mean threshold shifts (the scale of the y-axis of the left graph is multiplied by a factor of 10 compared to the right graph).

The means of the threshold shifts obtained during ipsi- and contra-lateral masking are displayed in Figure 7. A repeated measures ANOVA applied to ipsi-lateral masking resulted in a significant main effect for TS-TYPE (F (1, 9) = 127, p < 0.0001). No significant main effect for STIMULATION-SIDE and no significant interaction were found. The ipsi-lateral masking effect was stronger for PB. A repeated measures ANOVA applied to contra-lateral masking also resulted in a significant main effect for TS-TYPE (F (1, 9) = 12.6, p < 0.01), but neither a significant effect for STIMULUS-SIDE nor an interaction were found. The contra-lateral masking effects were far smaller than the ipsi-lateral masking effects; however, masking patterns were similar.

## Discussion

By using a complex stimulus design and by combining 40 Hz amplitude-modulated test stimuli of different spectral characteristics (PB and SB) with a comb-filtered noise (CFN) masker, in this study we were able to simultaneously examine both ASSR (primary auditory cortex origin) and N1m (non-primary auditory cortex origin). The results offer new insights into auditory neural interactions and hemispheric differences during simultaneous masking. These interactions depended on: (1) the frequency components of the stimuli, (2) level of the auditory system (peripheral or central), and (3) the hemisphere. There were two main findings. First, we observed that N1m and ASSR decrements during both ipsi- and contra-lateral masking depended on spectral differences between masker and test stimulus. Second, we obtained an inter-hemispheric difference for N1m response during both ipsi- and contra-lateral masking.

### Ipsi- and contra-lateral masking

In case of ipsi-lateral masking, both peripheral and central auditory pathways contribute to the masking effect. By contrast, in the case of contra-lateral masking, only neural interactions in the central auditory pathway contribute to the masking effect. Consequently, stronger masking effects should be expected for ipsi-lateral masking. Our MEG and behavioral results indeed confirm this hypothesis: significantly stronger masking effects on N1m and ASSR were found for ipsi-lateral compared to contra-lateral masking. During ipsi-lateral masking, displacements of the basilar membrane interact due to simultaneous presentation of TS and CFN, and such interactions along the basilar membrane are primarily responsible for N1m and ASSR decrements. However, overlapping basilar membrane displacements cannot explain the N1m and ASSR decrements observed during contra-lateral masking as masker and test sounds were presented to different ears. Therefore, we assume that these decrements were caused by neural interactions in the central auditory pathway. The significant ASSR decrements during contra-lateral masking imply that inhibitory neural interactions had already taken place in primary auditory cortex. However, it is possible that the central masking effects occurred at lower level, as anatomical studies in cat have shown that the superior olivary complex is the primary binaural interfering station [17].

### Influence of spectral components

N1m and ASSR decrements were more pronounced for PB compared to SB during both ipsi- and contra-lateral masking. In case of ipsi-lateral masking, these different decrements between PB and SB could be explained by the critical band concept suggested by Fletcher [13]. Those parts of the basilar membrane corresponding to the PB frequencies were already displaced by the continuous CFN overlapping in frequency content with the PB stimulus. This overlap resulted in smaller evoked responses elicited by PB compared to SB, which had less overlapping frequencies with the CFN. These findings support the results of a previous MEG study showing that N1m amplitude became smaller with increasing frequency overlap between masker and test tone [4].

During contra-lateral masking, binaural interactions between auditory neurons activated by left and right ear stimulations contributed to the significant N1m decrements. Our results showed significantly different N1m decrements between PB and SB. This result indicates that inhibitory neural interactions also depended on the frequency similarities between masker and test stimulus during contra-lateral masking. In accordance with previous psychoacoustical work [5], our MEG and behavioral data showed that the strongest masking effect was observed when a similar frequency sound was presented as masker to the opposite ear.

### Hemispheric differences

Normalized N1m decrements were significantly smaller in the left hemisphere during both ipsi- and contra-lateral masking. This result indicated that the left hemisphere might play a dominant role for sound processing in noisy environments. Recent neuroimaging studies [15,16] suggested a left hemispheric dominance for temporal and a right hemispheric dominance for spectral processing. The left hemispheric dominance for temporal processing can be considered as important during ipsi- and contra-lateral masking because temporal processing is essential for the segregation of target sounds from non-target sounds [18]. The temporal structure of the test signals might play an important role for sound detection. In the present study, test stimuli were characterized by a modulation envelope similar to a speech signal, whereas the masker was not. Hence, temporal cues might play an important role for the perception and the segregation of the test stimuli during both ipsi- and contra-lateral masking. Temporal cues are likely dominantly processed in the left hemisphere. That would lead to larger neural activities and smaller masking effects in the left hemisphere.

The hemispheric difference in auditory neural activities might be also explained by the 'asymmetric sampling in time' concept as suggested by Poeppel [19]. The author assumed that the left human auditory cortex has shorter temporal integration windows (25–50 ms), whereas the auditory cortex of the right hemisphere needs longer time windows (200–300 ms) to extract auditory information from the signal. An fMRI study [20] supported the hypothesis by demonstrating that the activities in the higher-order superior temporal sulci of left and right hemisphere depended on the modulation rate of the sound signals. Under natural circumstances, the quick analysis of deviant sound signals in noisy environments is essential for survival (i.e. the footfalls of predators). Therefore, the finer temporal resolution in the left hemisphere would play an important role for the monitoring and the detection of deviant sound signals in noisy situations. The results also showed that hemispheric differences during both ipsi- and contra-lateral masking were mainly caused by SB. Therefore, spectral cues seem to be helpful for the left hemisphere to separate signal from noise. This might indicate that spectral differences between signal and noise allow quick and rough segregation of sound signals in noisy environments in the left hemisphere by applying short temporal integration windows at the expense of slow and fine frequency analysis, which is dominantly accomplished in the right hemisphere. Thus, these results support the 'asymmetric sampling in time' concept by demonstrating that the masker affects the N1m response less in the left hemisphere compared to the right.

A previous MEG study [21] demonstrated that the N1m amplitude was significantly larger in the contra-lateral hemisphere, and another MEG study [22] showed right hemispheric laterality of the ASSR in addition to the effect of stimulation side. These results seem to be contradictory to the results obtained in the present study. Here, in order to clarify masking effects, we normalized N1m and ASSR source strengths during both ipsi- and contra-lateral masking with respect to the source strengths obtained in the control condition in left and right hemispheres individually. Hence, the contra-lateral N1m dominance and the right hemispheric ASSR laterality, which were obtained in the control condition, do not contribute to the hemispheric differences reported here.

### N1m decrements during contra-lateral masking

**Figure 8:**
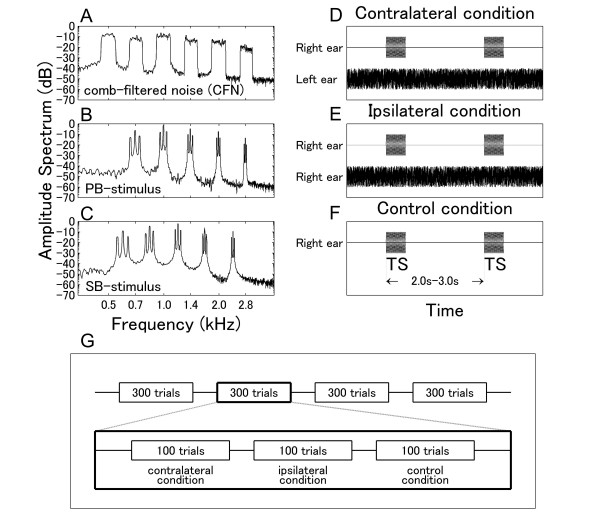
**Stimuli and experimental design**. Amplitude spectra of the auditory stimuli measured at the silicon earpiece fit to the subject's ear. (A) Spectrum of the masker (comb-filtered noise; CFN). The distance between neighboring centers of pass-band sections is half an octave in the frequency range between 0.5 and 2.8 kHz. (B) Spectrum of the pass-band stimulus (PB) composed of five spectral components corresponding to the center frequencies of the pass-band sections of the CFN. Due to the 40 Hz amplitude modulation, each component shows spectral peaks at its carrier frequency and at two sideband frequencies 40 Hz below and above. (C) Spectrum of the complex sound stimulus characterized by frequency components corresponding to the stop-band sections (SB) of the comb-filtered noise. All acoustical spectra reflect the low pass characteristic of the sound transmission system. Masking conditions. (D) Contra-lateral masking condition: TS and CFN were presented to different ears. (E) Ipsi-lateral masking condition: TS and CFN were presented to the same ear. (F) Control condition: Only the TS was presented to one ear, no masker was presented. Design of the experiment. (G) One session consisted of four blocks with three sub-blocks. Each sub-block consisted of either the contra-lateral, the ipsi-lateral, or the control condition. Within one session, the TS was presented only to the right or to the left ear.

We observed N1m decrements during contra-lateral masking, whereas in another MEG study [9] this effect did not reach significance. Several explanations for this apparent discrepancy appear conceivable: first, in the previous study [9], a shorter time interval between stimulus onsets was used (0.8–1.0 s). Our longer time interval of 2.0–3.0 s is expected to result in larger N1m source strengths, which in turn might have lead to the observed higher sensitivity of the N1m decrements during contra-lateral masking. Second, we used complex stimuli consisting of several frequency components (Figure 8B,C) instead of the pure tone used in the previous MEG study [9]. These comparably complex stimuli might have also contributed to the increment in N1m source strength, because complex sounds activate more neurons in non-primary auditory cortex than pure tones [23]. Third, we used a CFN composed of narrowly defined band-passed noises as masker. Compared to the broadband noise used in the previous MEG study [9], the CFN might selectively have activated neurons that correspond to the pass-band frequencies of the CFN. Such frequency specific neural activities elicited by the contra-laterally presented CFN might have more efficiently contributed to the significant N1m decrements we have observed here. Fourth, in the present study, we used amplitude-modulated sounds characterized by specific temporal structures causing temporal encoding corresponding to the stimulus envelopes. Temporal neural activities elicited by the amplitude-modulated sounds might have caused the significant N1m decrements observed in the present study, as Galambos and Makeig [6] showed significant contra-lateral masking effects by presenting a sound with a specific temporal feature.

### Right ear advantage

ASSR decrements were significantly different between ears during ipsi-lateral masking: decrements were more pronounced for left compared to right ear stimulation. The less pronounced ASSR decrements during ipsi-lateral masking might reflect the so-called 'right ear advantage' suggested by a previous study [24]. The author proposed that the right ear advantage was due to the amount of efferent inhibition, which is relatively small in right compared to left ear stimulation. Thus, the smaller number of inhibitory efferent neurons in the right ear might cause the less pronounced ASSR decrements during ipsi-lateral masking.

## Conclusion

In the present study, we have observed smaller N1m decrements in the left hemisphere during both ipsi- and contra-lateral masking. This suggests left hemispheric dominance for auditory processing in noisy environments. As test stimuli, we used amplitude-modulated sounds characterized by 'fine structure' and 'envelope' at the same time. These sounds are similar to speech signals, but do not carry speech information [25]. Therefore, our results may be interpreted as reflection of basic hemispheric specialization contributing to higher-level auditory analysis such as speech processing in noisy environments.

## Methods

### Subjects

Ten subjects (two females; mean age 29 years, range 19–37 years) with no history of otological or neurological disorders participated in the present study. All subjects were strongly right-handed (assessed via "Edinburgh Handedness Inventory" [26]). They had normal hearing thresholds within the frequency range of 250 and 8000 Hz as tested by means of clinical pure tone audiometry. The subjects consented for participation after having been informed about the nature of the study. The Research Ethics Board of Baycrest Centre approved all experimental procedures, which were in accordance with the Declaration of Helsinki.

### Masker and test stimuli

To investigate neural interactions between similar and different frequencies, we used a comb-filtered noise (CFN) derived from white noise by applying multiple band-pass filters with widths of a quarter of an octave as masker (Figure 8A) and two kinds of 40 Hz amplitude-modulated complex tones as test stimuli (TS). The first TS was a pass-band stimulus (PB) composed of five spectral components corresponding to the centers of the pass-band sections (0.7, 1.0, 1.4, 2.0, 2.8 kHz) of the CFN (Figure 8B). The second TS was a stop-band stimulus (SB) containing the center frequencies of the stop-band sections (0.59, 0.83, 1.19, 1.66, and 2.39 kHz) of the CFN (Figure 8C). Both TS with duration of 500 ms were 100% amplitude modulated with a 40 Hz sinusoid. To examine possible sound distortions due to the sound delivery system, we measured the amplitude spectra of the CFN and the TS at the earpiece; the results are displayed in Figure 8A–C. The simultaneous presentation of CFN and TS allowed the examination of interactions between auditory neurons activated by either similar (PB) or different frequencies (SB) in primary as well as non-primary auditory cortices [27,28].

### Experimental design

Three experimental conditions were applied: contra-lateral masking, ipsi-lateral masking, and no masking control condition (Figure 8D–F). Two sessions were performed for each subject. Within each session, the TS were presented either to the left or to the right ear. The CFN masker was presented either to the contra-lateral ear (Figure 8D) or to the ipsi-lateral ear (Figure 8E) in each session. No CFN was presented in the control condition (Figure 8F). Hence, the ear of TS presentation did not differ between ipsi-lateral and contra-lateral masking conditions within a session, but the masker presentation sides did. The time interval between stimulus onsets was randomized between 2.0 and 3.0 s. A schematic illustration of the time course of a session is given in Figure 8G. Three blocks containing 100 trials for each masking condition were repeated four times in one session in randomized order. We adopted a block design to avoid masker-onset effects.

Both CFN and TS were presented at 45 dB SL (sensation level) in order to avoid interaural cross talk. The intensities of stimuli and masker were individually adjusted at the beginning of each experimental session. TS were prepared as sound files and presented via STIM software (NeuroScan Inc., Charlotte, NC, USA) using ER30 transducers (Etymotic Research, Elk Grove Village, IL, USA), plastic tubes of 2.5 m length and silicon ear pieces fitting to the subject's ears. The CFN was played from a CD player and superimposed on the TS.

### Data acquisition

Auditory evoked magnetic fields were recorded with a helmet-shaped 151-channel whole cortex neurogradiometer (OMEGA, CTF Systems, VSM MedTech Inc., Coquitlam, British Columbia, Canada) in a quiet, magnetically shielded room. The subjects were seated comfortably and watched a silent movie of their choice during the MEG measurement in order to keep them in a stable alert state. The magnetic field signals were 200 Hz low-pass filtered online and sampled at a rate of 625 Hz.

### Data analysis

Epochs of magnetic field data starting 300 ms before the onset of the TS and ending 200 ms after the offset of the TS (in total: 1.0 s) were averaged after artifact rejection (threshold: 3.0 picotesla). After 30 Hz low-pass filtering and baseline correction based on the 300 ms pre-stimulus interval, spatiotemporal equivalent current dipoles (ECDs; one for each hemisphere) were estimated for the averaged magnetic field distribution of the N1m response in the control condition. A 10 ms time window prior to the maximal global field power, measured as root mean square across all sensors around 100 ms after TS onset, was used for estimation of the transient N1m source. ECD locations and orientations in each hemisphere were determined in a head based Cartesian coordinate system with the origin set to the midpoint of the medial-lateral axis (y-axis) between the entrances of the left and right ear-canals: the posterior-anterior axis (x-axis) ran between nasion and the origin; the inferior-superior axis (z-axis) ran through the origin perpendicularly to the x-y-plane.

The source analysis resulted in two estimates for the N1m sources in the right and left hemispheres. Based on the ECD coordinates and orientations obtained in the control condition, the method of source space projection was applied to the averaged magnetic field of each condition. This method combines the magnetic field waveforms obtained from each sensor weighted by the sensitivity of each sensor for a source at the specified location into a single waveform of the dipole moment. ASSR was analyzed in a similar way. The model of ECD was applied to the averaged magnetic field data (band-pass filtered between 30 and 50 Hz) within the 300 ms time interval starting 200 ms after TS onset until the end of the stimulus. N1m and ASSR source strength decrements observed in ipsi- or the contra-lateral masking conditions were normalized with respect to the source strength obtained in the control condition for each hemisphere of each subject (normalized decrement = (source strength unmasked-source strength masked)/source strength unmasked). Those normalized decrements are then interpreted as masking effects between 0 (no masking) and 1 (complete extinction). In order to evaluate the normalized N1m and ASSR decrements, repeated measures analyses of variance (ANOVAs) were performed to evaluate three factors (STIMULUS-SIDE × TS-TYPE × HEMISPHERE) for ipsi- and contra-lateral masking independently. The source locations of N1m and ASSR were analyzed by paired t-test with respect to each axis in each hemisphere. P values smaller than 0.05 were accepted as significant.

### Behavioral measurement

In addition, we carried out psychoacoustical measurements to assess the threshold shifts during ipsi- and contra-lateral masking. Ten right-handed subjects (four females, mean age 28 years, range 24–32 years) with no history of otological or neurological disorders participated in the behavioral study. All measurements were performed in an acoustically shielded room. As the sound signals used for the MEG measurements were low-pass filtered due to the transfer characteristic of the sound delivery system, we used the CFN and the TS recorded at the earpiece in the magnetically shielded room for the behavioral measurements in order to make behavioral measurement and MEG measurement comparable. Thus, the sound signals used for MEG and behavioral measurements had identical amplitude spectra (Figure 8A–C). The TS were prepared as sound files and presented via audiometer (AA-71, Rion Co. Ltd., Tokyo, Japan) by means of headphones. The CFN was played from a CD player and superimposed on the TS under control of the audiometer. We first measured the threshold for the CFN for each ear (left or right, in random order) individually and then we measured the thresholds for PB and SB for ipsi-lateral, contra-lateral and control conditions (order randomized between subjects). Sensitivity evaluations were made in 1 dB steps. The CFN was presented at the intensity level of 45 dB SL during ipsi-lateral and contra-lateral masking. To evaluate threshold shifts, repeated measures ANOVAs were calculated using two factors (STIMULUS-SIDE × TS-TYPE) for ipsi-lateral and contra-lateral masking.

## Authors' contributions

HO conceived of the study, HO and CP designed the experimental setup. HO acquired the data and drafted the manuscript. All authors participated the data evaluation and interpretation and approved the final version of the manuscript.
